# Topographically Distinct Projection Patterns of Early-Generated and Late-Generated Projection Neurons in the Mouse Olfactory Bulb

**DOI:** 10.1523/ENEURO.0369-20.2020

**Published:** 2020-12-02

**Authors:** Uree Chon, Brandon J. LaFever, Uyen Nguyen, Yongsoo Kim, Fumiaki Imamura

**Affiliations:** 1 Department of Neural and Behavioral Sciences, Penn State College of Medicine, Hershey, PA 17033; 2 Department of Pharmacology, Penn State College of Medicine, Hershey, PA 17033

**Keywords:** axonal projection, development, mitral cell, neuronal birthdate, olfactory bulb

## Abstract

In the mouse brain, olfactory information is transmitted to the olfactory cortex via olfactory bulb (OB) projection neurons known as mitral and tufted cells. Although mitral and tufted cells share many cellular characteristics, these cell types are distinct in their somata location and in their axonal and dendritic projection patterns. Moreover, mitral cells consist of heterogeneous subpopulations. We have previously shown that mitral cells generated at different embryonic days differentially localize within the mitral cell layer (MCL) and extend their lateral dendrites to different sublayers of the external plexiform layer (EPL). Here, we examined the axonal projection patterns from the subpopulations of OB projection neurons that are determined by the timing of neurogenesis (neuronal birthdate) to understand the developmental origin of the diversity in olfactory pathways. We separately labeled early-generated and late-generated OB projection neurons using *in utero* electroporation performed at embryonic day (E)11 and E12, respectively, and quantitatively analyzed their axonal projection patterns in the whole mouse brain using high-resolution 3D imaging. In this study, we demonstrate that the axonal projection of late-generated OB projection neurons is restricted to the anterior portion of the olfactory cortex while those of the early-generated OB projection neurons innervate the entire olfactory cortex. Our results suggest that the late-generated mitral cells do not extend their axons to the posterior regions of the olfactory cortex. Therefore, the mitral cells having different birthdates differ, not only in cell body location and dendritic projections within the OB, but also in their axonal projection pattern to the olfactory cortex.

## Abstract (precis)

In the mouse brain, olfactory information is transmitted to the olfactory cortex via olfactory bulb (OB) projection neurons known as mitral and tufted cells. Although mitral and tufted cells share many cellular characteristics, these cell types are distinct in their somata location and in their axonal and dendritic projection patterns. Moreover, mitral cells consist of heterogeneous subpopulations.

## Significance Statement

The olfactory bulb (OB) contains long-range projection neurons with distinct connectivity to higher order brain regions. Here, we examined how the birthdate of the OB projection neurons correlates to the generation of differential connectivity patterns. We used *in utero* electroporation and high-resolution 3D imaging of the whole mouse brain, and determined the topographically distinct axonal projection patterns of early-generated and late-generated OB projection neurons. Our results show that the timing of neurogenesis is a determining factor for the innervation of OB projection neurons and indicate that mitral cells having different birthdates are the origins of distinct olfactory information pathways. Our study provides novel insights into the formation of neuronal circuits processing multiple aspects of olfactory information.

## Introduction

The olfactory bulb (OB) is the first relay station for olfactory information in the vertebrate central nervous system. Within the OB, projection neurons, mitral and tufted cells, receive input from olfactory sensory neurons and transmit the olfactory information further to the olfactory cortex consisting of several brain regions. Accumulating evidence suggests that distinct regions within the olfactory cortex process different aspects of the olfactory information. For example, the piriform cortex (PIR) is critical for odor discrimination, identification, and memory (Choi et al., 2011; Wilson and Sullivan, 2011; Bekkers and Suzuki, 2013; Blazing and Franks, 2020), the anterior olfactory nucleus (AON) contributes to odor source detection (Kikuta et al., 2010; Liu et al., 2020), the olfactory tubercle (OT) has close interaction with a reward system (Ikemoto, 2007; Wesson and Wilson, 2011; Gadziola et al., 2015; Yamaguchi, 2017; Zhang et al., 2017), and the amygdala mediates the fear responses induced by predator odors (Root et al., 2014; Isosaka et al., 2015; Kondoh et al., 2016). The segregation of the neural pathways controlling these behavioral responses likely begins with diverse subpopulations of OB projection neurons (Sosulski et al., 2011; Bear et al., 2016).

Historically, the major criterion to discriminate between mitral and tufted cells is somata location within the OB. However, an increasing number of studies have reported differences in the morphologic and physiological properties between these two types of projection neurons in the mammalian OB (Igarashi et al., 2012; Adam et al., 2014; Nagayama et al., 2014b; Cavarretta et al., 2018). In particular, mitral and tufted cells project their axons to distinct regions in the olfactory cortex. While a single mitral cell innervates almost the entire olfactory cortical areas, tufted cells project axons only to the anterior portion of the olfactory cortex, including the OT and AON (Nagayama et al., 2010; Igarashi et al., 2012; Hirata et al., 2019). This suggests that different aspects of olfactory information are processed in parallel pathways originating from mitral and tufted cells. In addition, recent studies have shown that mitral cells consist of heterogeneous subpopulations with different cellular properties. Although mitral cells typically extend their secondary dendrites in the deep sublayer of the external plexiform layer (EPL), some mitral cells extend their secondary dendrites in the superficial sublayer of the EPL (Mori et al., 1983; Orona et al., 1984; Mouradian and Scott, 1988). The diversity of intrinsic biophysical properties among mitral cells, such as interspike interval, firing frequency, and the I_h_ sag current, have also been reported (Nagayama et al., 2004; Padmanabhan and Urban, 2010; Angelo et al., 2012; Igarashi et al., 2012). These differences in molecular and biophysical properties may endow mitral cells with different odor response properties (Dhawale et al., 2010; Kikuta et al., 2013). However, a critical question of whether different subsets of mitral cells project axons to different regions in the olfactory cortex has yet to be answered.

In the developing mouse main OB, mitral cells are generated between embryonic day (E)9 and E13, which is earlier than tufted cell birthdates (Hinds, 1968; Blanchart et al., 2006; Imamura et al., 2011). We previously showed that early-generated and late-generated mitral cells were preferentially localized at the dorsomedial and ventrolateral portion of the mitral cell layer (MCL), respectively (Imamura et al., 2011). Furthermore, we separately labeled subsets of mitral cells with different birthdates using the *in utero* electroporation method and revealed that early-generated and late-generated mitral cells extend their lateral dendrites in the deep and superficial EPL, respectively, (Imamura and Greer, 2015b). It has been speculated that neuronal birthdates may also control the axonal projection patterns of OB projection neurons to the olfactory cortex (Imamura et al., 2011; Hirata et al., 2019). These previous studies demonstrated that the OT receives axonal inputs preferentially from tufted and late-generated mitral cells (Scott et al., 1980; Imamura et al., 2011), and segregated axonal projections are formed by early-generated mitral cells and late-born external tufted cells (Hirata et al., 2019). Nevertheless, the axonal projection of late-generated mitral cells to the olfactory cortex other than the OT, and differences in axonal projection patterns between early-generated and late-generated mitral cells have not yet been elucidated. In this study, we separately labeled the early-generated and late-generated OB projection neurons using the *in utero* electroporation method and quantitatively analyzed axonal projection patterns in the whole mouse brain using serial two-photon tomography (STPT) imaging. Our study demonstrates that the axonal projection patterns of tufted cells as well as late-generated mitral cells are restricted to the anterior portion of the olfactory cortex.

## Materials and Methods

### Animals

The offspring of CD1 female mice (Charles River; strain code 022; RRID:IMSR_CRL:022) mated with the Tbx21-Cre (B6;CBA-Tg (Tbx21-cre)1Dlc/J; The Jackson Laboratory; stock #024507; RRID:IMSR_JAX:024507; Haddad et al., 2013) or Tbx21Cre x tdTomato male mice were used for the *in utero* electroporation in this study. The Tbx21Cre x tdTomato line was created by crossing Tbx21-Cre mice with B6.Cg-Gt(ROSA)26Sortm9 (CAG-tdTomato) Hze/J reporter mice (The Jackson Laboratory; stock #007909; RRID:IMSR_JAX:007909; Nguyen and Imamura, 2019). The day on which we found a copulation plug was called E0, and the succeeding days of gestation were numbered in order. All protocols were approved by, and all methods were performed in accordance with the guidelines of the Institutional Animal Care and Use Committee (IACUC) of Penn State College of Medicine.

### *In utero* electroporation

The plasmid that drives the expression of a GFP gene under the CAG promoter in the presence of Cre recombinase (pCALNL-GFP; RRID:Addgene_13770) and the plasmid that expresses tdTomato fluorescent protein under the CAG promoter (pCAG-tdTomato; RRID:Addgene_83029) were obtained from Addgene. *In utero* electroporation was performed in accordance with the procedure as previously reported (Imamura and Greer, 2013, 2015a). Briefly, pregnant female mice were anesthetized with an intraperitoneal injection of ketamine (100 mg/kg) and xylazine (10 mg/kg), and the uterine horns were carefully taken out from the abdominal cavity. Approximately 0.5 μl of DNA solution (1.5–2.5 μg/μl in 5 mM Tris-HCl (pH 8.0) and 0.5 mM EDTA) was injected into the lateral cerebral ventricle of embryos by insertion of a glass pipette. The DNA solution was mixed with 200 μg/ml of Fast Green for visible confirmation of the injection site. Then, electroporation was conducted by applying square electric pulses: two pulses of 30 V, 50-ms duration with a 950-ms interval. To efficiently label the mitral cell precursors in the presumptive OB, a positive current was applied from posterior to anterior. Upon completion of the electroporation, the uterine horns were repositioned in the abdominal cavity. Following suturing, the animals were allowed to recover in a warm environment and returned to their home cage. The animals were given a subcutaneous injection of Carprofen (5 mg/kg) for pain relief before and after the surgery.

### Immunohistochemistry

Postnatal day (P)7 pups were killed by decapitation and fixed in 4% paraformaldehyde (PFA) overnight. The fixed brains were cryopreserved in 30% sucrose (wt/vol) in PBS and embedded in optimal cutting temperature compound (Sakura Finetek USA). The olfactory tissues were cut on a cryostat into 20-μm slices, collected on Superfrost Plus Micro Slides (Avantor) and stored at −80°C until use. The slices were pretreated for 30 min in 0.025 m HCl at 65°C and rinsed with 0. 1 m borate buffer (pH 8.5), PBS and TBS-T [10 mm Tris-HCl (pH 7.4), and 100 mm NaCl with 0.3% Triton X-100 (v/v)]. The slices were then blocked with blocking buffer [5% normal donkey serum (v/v) in TBS-T] at 20–25°C for 1 h and incubated with primary antibodies, chicken anti-GFP (1:1000; Abcam catalog #ab13970, RRID:AB_300798) and rabbit anti-tdTomato (1:200; Rockland Immunochemicals catalog #600-401-379, RRID:AB_2209751), diluted in blocking buffer overnight at 4°C. Sections were washed with TBS-T and then incubated with secondary antibodies, donkey anti-chicken IgY conjugated with Cy2 (1:200; Jackson ImmunoResearch catalog #703-225-155, RRID:AB_2340370), and donkey anti-rabbit IgG conjugated with Alexa Fluor 555 (1:300; Thermo Fisher Scientific catalog #A-31572, RRID:AB_162543), with 4’6-diamino-2-phenylindole dihydrochloride (DAPI; D1306; Thermo Fisher Scientific; RRID:AB_2629482) for nucleus staining for 1 h. The immunoreacted sections were washed and coverslipped with Fluoro-Gel mounting medium (Electron Microscopy Science).

### STPT imaging and data analysis

Mice were transcardially perfused with 0.9% saline and 4% PFA. The dissected brains were fixed in 4% PFA at 4°C overnight. These brains were stored in 0.05 m phosphate buffer (PB) at 4°C until imaging. Detailed information about STPT imaging and analysis were previously described (Jeong et al., 2016; Newmaster et al., 2020). Briefly, the brain samples were embedded in oxidized 4% agarose and cross-linked by 0.05 m sodium borohydride for imaging preparation. This agarose block with an embedded sample was placed in a buffer chamber filled with 0.05 m PB for imaging. We used Tissuecyte 1000 (TissueVision) to perform serial two-photon tomography imaging (Ragan et al., 2012). Each brain was imaged in the coronal plane with a two-photon laser (Coherent UltraII) at 910 nm with a 560-nm dichroic mirror to acquire both green and red spectrum signals. Images were acquired as 280 serial sections (12 × 16 *xy* tiles, 700 × 700 pixels field of view, 1 × 1 μm *xy* resolution) at every 50 μm in thickness. Using a custom-built algorithm, the images were reconstructed and the projection pattern was analyzed. To detect the GFP projection signal, both signal (green) and background (red) images were normalized by z-normalization. Then, the normalized signal channel was subtracted by the normalized background channel. This procedure helped to remove background regardless of the background brightness. Signals from the subtracted images were binarized using a threshold (eight times of SD from the signal channel). The binarized signal was counted in each evenly spaced and non-overlapping rectangular voxel (20 × 20 × 50 μm^3^) across the whole brain. This procedure helped to quantify the projection area in the brain. Then, each brain with projection signals was registered to Allen common coordinate framework (CCF; Wang et al., 2020) using Elastix (Klein et al., 2010) with previously defined affine and b-spline parameters at 20 × 20 × 50 μm *xyz* resolution (Kim et al., 2017).

To quantify the ratio of GFP+ mitral and tufted cells in the main OB, we first selected images of five coronal slices taken every 600 μm from anterior to posterior in each OB. Brightness levels were adjusted in Photoshop software (Adobe) to allow for sufficient visualization, but the images were otherwise unaltered. Next, we manually counted all mitral cells classified as GFP+ cell bodies in the MCL, and tufted cells classified as GFP+ cell bodies in the EPL, in each slice. The ratio of GFP+ mitral cells to GFP+ tufted cells was calculated by dividing the total number of mitral cells counted from five slices by that of tufted cells. The values were acquired from six OBs (five mice) and eight OBs (seven mice) electroporated at E11 and E12, respectively.

### Olfactory area flatmap

One OB from each mouse was used to generate a flatmap (*n* = 5 for IUE@E11 and *n* = 7 for IUE@E12). First, we generated a maximum projection pattern using the “Add” function on Fiji (ImageJ, NIH) using registered signals onto the reference brain. Then, the lateral olfactory cortex/cortical plate areas with projection signals were selected and exported out using the “TrakEM2” function on Fiji. The exported region was divided into evenly spaced bins to generate a flatmap in the adult reference brain. Each region was given a specific numerical value as a regional ID. To quantify projection signals on the flatmap drawn on the reference brain, GFP signals in each flatmap bin were quantified. Densities of projection signals were measured by counting the numbers of GFP-positive pixels and total pixels in each bin; the quantifications are represented in percentages of GFP-positive pixels. The density was plotted on the flatmap using Excel (Microsoft) and Illustrator (Adobe).

## Results

### Electroporation of plasmid vectors to the OB projection neurons

**Figure 1.**
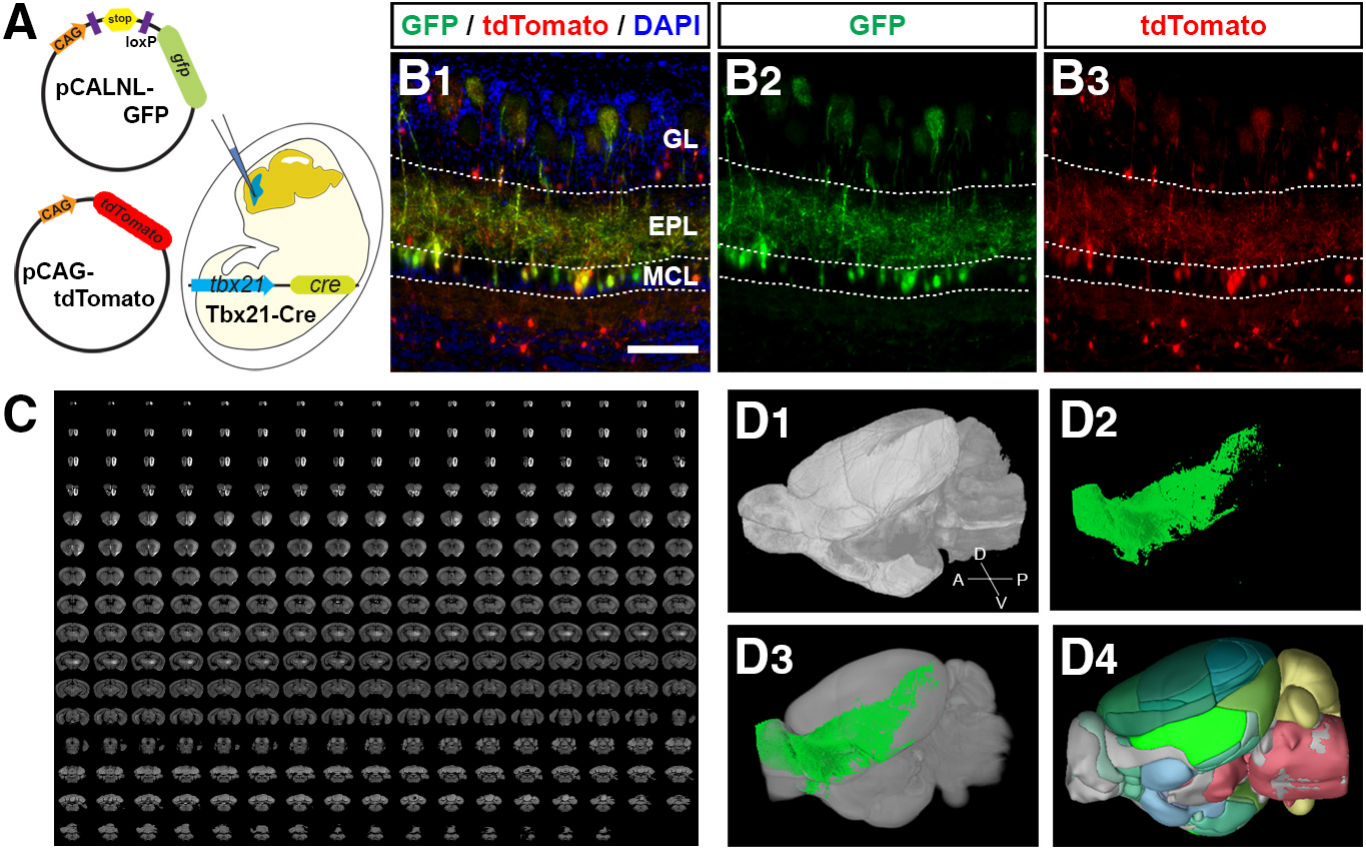
Strategy to analyze the axonal projection patterns of OB projection neurons. ***A***, Schematic diagram of *in utero* electroporation. Plasmid mixture was injected into the lateral ventricle of the mice embryos, and the negative current was applied from posterior to anterior to electroporate the cells in the presumptive OB. ***B***, Medial region of a coronal section of P7 Tbx21-Cre OB electroporated with pCALNL-GFP and pCAG-tdTomato, at E11. OB projection neurons, mitral and tufted cells, express both GFP (green) and tdTomato (red) while tdTomato+ interneurons are negative for GFP. All nuclei were stained with DAPI (blue). Scale bar: 100 μm. ***C***, 270 serial section images acquired in STPT. ***D***, 3D reconstruction from the SPTP imaging (***D1***), axonal projection signal (***D2***), registered axonal signals in Allen CCF reference brain (***D3***), and anatomic labels in the reference brain (***D4***).

We previously showed that *in utero* electroporation performed at E10 and E12 preferentially labeled early-generated and late-generated OB projection neurons, respectively (Imamura and Greer, 2015b). However, the electroporation also delivers the plasmids into some interneurons in the OB as well as neurons in the other brain regions including the AON, OT, and PIR, which makes it difficult to analyze the axonal projection patterns of OB projection neurons to the olfactory cortex. To overcome this difficulty, we used the Tbx21-Cre transgenic mice in which the Cre recombinase expression is controlled by the Tbx21 promoter (Haddad et al., 2013; Nguyen and Imamura, 2019). Since Tbx21 is exclusively expressed by OB projection neurons in the mouse brain (Mitsui et al., 2011), this method ensures that GFP expression will occur only in OB projection neurons by electroporating the plasmid, pCALNL-GFP, which expresses GFP on the presence of Cre recombinase (Fig. 1*A*). When the pCALNL-GFP and pCAG-tdTomato plasmids are simultaneously electroporated into the Tbx21-Cre mice brain at E11, fluorescent signals of tdTomato were seen in all neuronal cell types, while GFP signals were restricted to the mitral and tufted cells in the OB at P7 (Fig. 1*B*).

### Segregated labeling of OB projection neurons based on their birthdates

To compare the axonal projection patterns of OB projection neurons generated at different developmental stages, we electroporated pCALNL-GFP into the brains of Tbx21Cre x tdTomato transgenic mice. In these mice, tdTomato is expressed by all OB projection neurons (Nguyen and Imamura, 2019). Our previous studies showed that differences in cell body location and dendrite extension patterns between E11-generated and E12-generated mitral cells were greater than those between E10-generated and E11-generated mitral cells (Imamura et al., 2011; Imamura and Greer, 2015b). We formed the assumption that E12-generated mitral cells significantly change their cellular properties from E11-generated mitral cells. Therefore, we conducted *in utero* electroporation labeling on E11 (IUE@E11) and E12 (IUE@E12) to examine whether there is a birthdate-dependent difference in the axonal projection patterns. In this experiment, the electroporated mice were killed between six and eight weeks old (P42–P53). The GFP signals from the OB projection neurons were examined and analyzed throughout the whole brain at cellular resolution using STPT and custom-built data processing pipeline (for more details, see Materials and Methods; Fig. 1*C*,*D*; Jeong et al., 2016).

**Figure 2.**
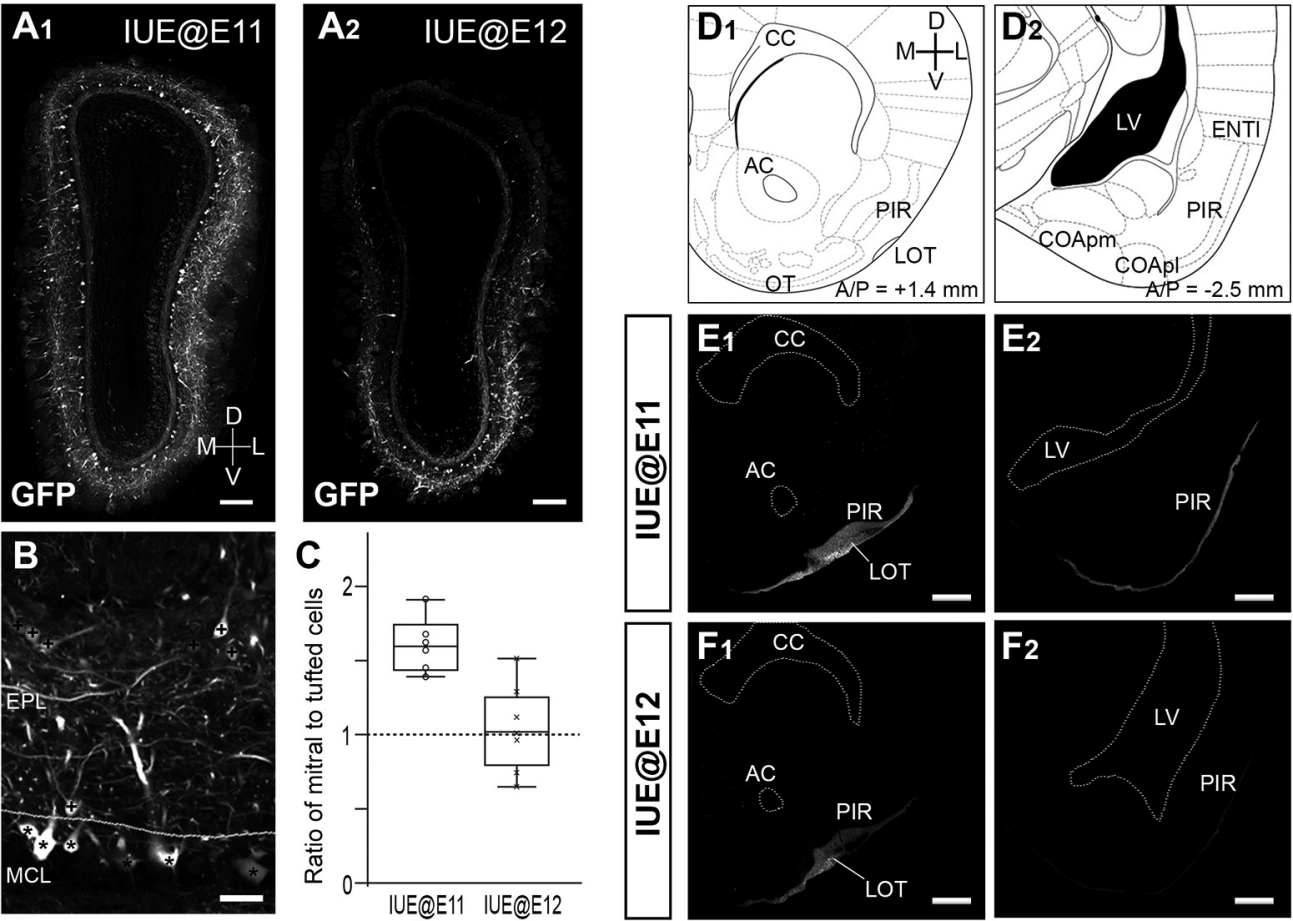
Labeling of different subpopulations of OB projection neurons using *in utero* electroporation. ***A***, Coronal sections of the OBs from adult mice in which electroporations were performed at E11 (***A1***) or E12 (***A2***). GFP is expressed only in mitral and tufted cells. IUE@E12 preferentially labeled mitral cells in the ventrolateral part of the OB. ***B***, Quantification of mitral and tufted cells in the OB. Cells that have GFP+ somata in the MCL and EPL were defined as mitral cells (marked with asterisks) and tufted cells (marked with plus signs), respectively. ***C***, Ratios of mitral cells to tufted cells calculated from IUE@E11 (*n* = 5) and IUE@E12 (*n* = 7) OBs are shown with box plots. ***D–F***, Projection of GFP+ axons to the anterior (***D1***, ***E1***, ***F1***) and posterior (***D2***, ***E2***, ***F2***) part of the olfactory cortex in the IUE@E11 (***E***) and IUE@E12 brain (***F***). Reference brain regions observed in ***E***, ***F*** are cited from a mouse brain atlas (Paxinos and Franklin, 2001). GFP+ axons are seen in the anterior PIR of both IUE@E11 (***E1***) and IUE@E12 (***F1***) brains, whereas only the IUE@E11 brain has a significant GFP signal in the posterior PIR (***E2***, ***F2***). Scale bars: 200 μm (***A***), 50 μm (***B***), and 500 μm (***E***, ***F***). EPL: external plexiform layer; MCL: mitral cell layer; CC: corpus callosum; AC: anterior commissure; LOT: lateral olfactory tract; PIR: piriform cortex; OT: olfactory tubercle; LV: lateral ventricle; COApl and COApm: posterolateral and posteromedial cortical amygdala; ENTl: lateral entorhinal cortex.

Figure 2*A* shows the OBs of IUE@E11 and IUE@E12 mice. To examine how the plasmid was taken up between mitral and tufted cells, the number of GFP+ mitral cells and tufted cells were counted separately in each OB (Fig. 2*B*). Here, we should note that displaced mitral cells, sometimes called internal tufted cells, located at the border of the MCL and EPL were included in the population of mitral cells (Nagayama et al., 2014a). By calculating the ratios of GFP+ mitral cells to tufted cells, we confirmed that a significant number of mitral cells was labeled with GFP in the OBs of both IUE@E11 mice (1.60 ± 0.13; *n* = 6) and IUE@E12 mice (1.05 ± 0.20; *n* = 8), although the proportion of labeled mitral cells was lower in the IUE@E12 (Fig. 2*C*). Of particular note is that GFP+ mitral cells were preferentially found in the ventrolateral MCL of the IUE@E12 mice whereas the GFP+ mitral cells are distributed throughout the whole MCL of the IUE@E11 mice (Fig. 2*A*). We also confirmed that the GFP+ secondary dendrites were preferentially distributed in the superficial EPL in the IUE@E12 OB. These are consistent findings with our previous study (Imamura and Greer, 2015b) and suggest that, among mitral cells, the late-generated mitral cells were predominantly labeled in the IUE@E12 OB.

### Different axonal projection patterns between early-generated and late-generated OB projection neurons

Upon imaging the GFP signals in the olfactory cortex, strong signals were observed in the anterior regions, including the lateral olfactory tract (LOT) and the anterior PIR, of both IUE@E11 and IUE@E12 brains (Fig. 2*D1*,*E1*,*F1*). In contrast, IUE@E11 brains showed stronger GFP signals compared with the IUE@E12 brains in the posterior regions of the olfactory cortex, such as the posterior PIR and lateral entorhinal cortex (ENTl; Fig. 2*D2*,*E2*,*F2*). This finding suggests that early-generated OB projection neurons project to broader olfactory cortical areas than the late-generated neurons.

**Figure 3.**
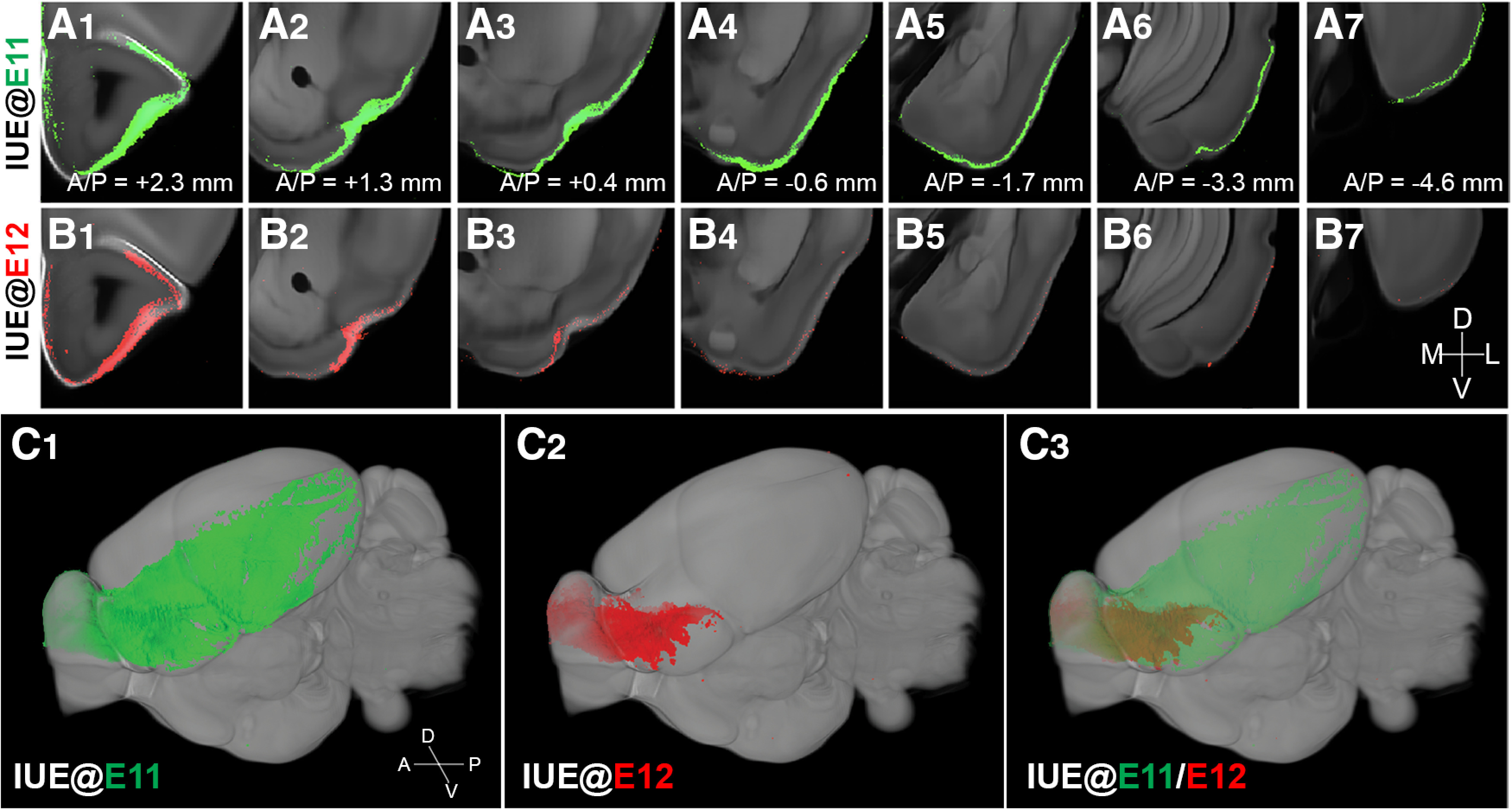
Brain-wide axonal projection pattern from OB neurons with different birthdates. ***A***, ***B***, Axonal projection signals from IUE at E11 (***A***) and IUE at E12 (***B***) registered on the reference brain. GFP signals were pseudo-colored as red in ***B*** to facilitate a comparison between signals from two different birth dates. Bregma anterior/posterior (A/P) coordinates were included. ***C***, 3D rendering of axonal projection from IUE at E11 (***C1***), E12 (***C2***), and merged (***C3***) in the reference brain. Late-generated OB projection neurons labeled with IUE@E12 do not project their axons to the posterior regions of the olfactory cortex.

To further analyze the long-range axonal projection patterns of OB projection neurons, GFP signals observed above the threshold level were overlaid onto the coronal sections of a reference brain. Figure 3*A*,*B* depicts the distribution of GFP signal in the olfactory cortex from anterior to posterior imaged from a representative IUE@E11 (mitral/tufted ratio = 1.45) and IUE@E12 mouse brain (mitral/tufted ratio = 0.96; pseudo-colored as red for easy comparison), respectively. In the IUE@E11 brain, the GFP signals were seen in almost every region within the olfactory cortex (Fig. 3*A*). In contrast, the GFP signal was observed only in the anterior portion of the brain in the IUE@E12 (Fig. 3*B*). The difference in the distribution of GFP+ axons between IUE@E11 and IUE@E12 brains was clearly displayed when signals from IUE@E11 (green) and IUE@E12 (red; pseudo color) were overlaid onto the reference sections and visualized in a skewed 3D angle (Fig. 3*C*). These results demonstrate that a subset of OB projection neurons generated at around E12 restrict their axonal projections solely to the anterior regions of the olfactory cortex.

**Figure 4.**
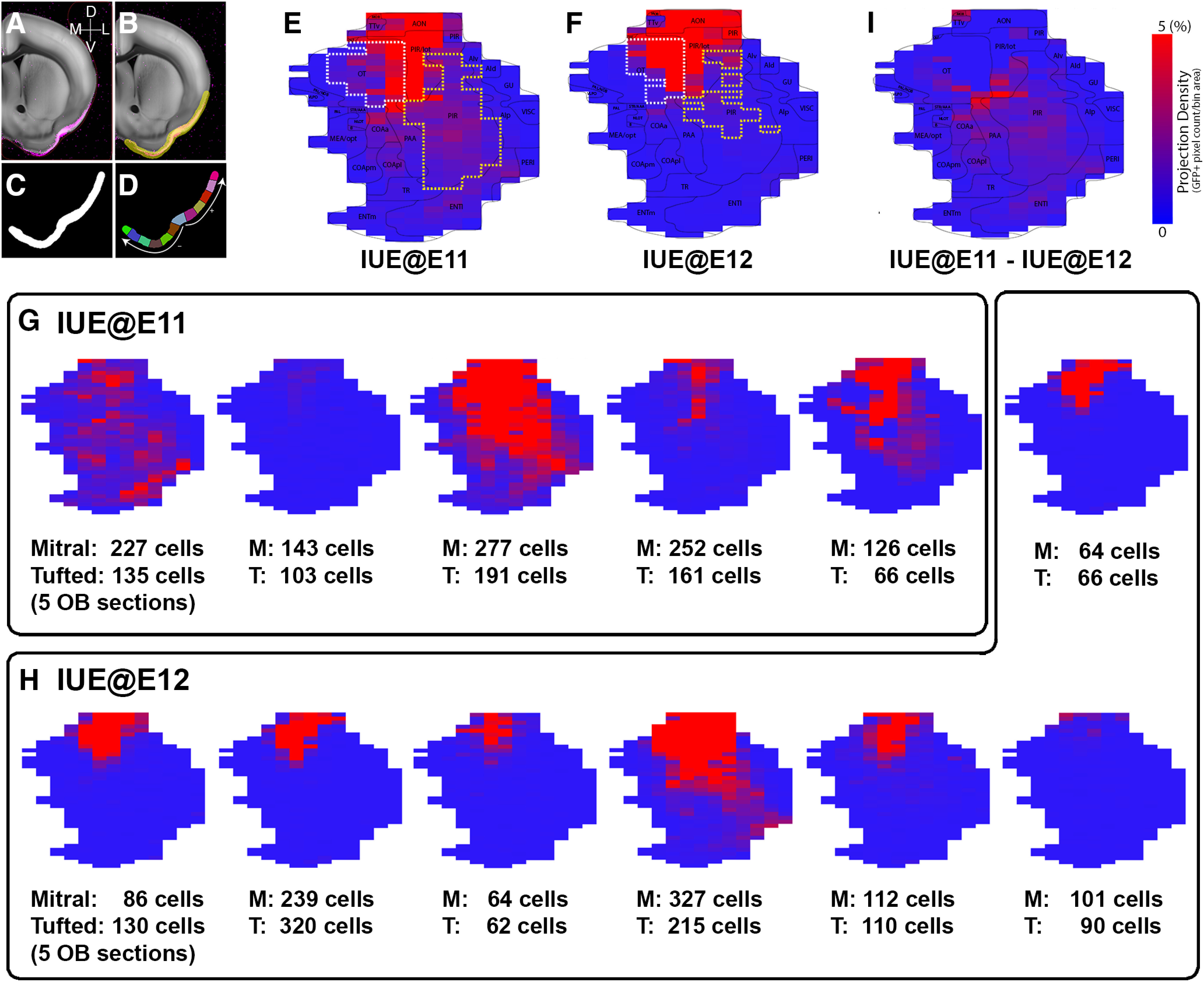
Topographical axonal projection pattern on 2D flatmap. ***A–D***, Creation of 2D flatmap. Axonal projection signal in the reference brain (***A***) and binary mask to cover areas with projection signal (***B***), binary mask (***C***), and evenly spaced bins (***D***) to create the flatmap (for details, see Materials and Methods). ***E***, ***F***, Averaged axonal projection signal in heatmap from IUE at E11 (***E***) and E12 (***F***). Bins that show >5% of GFP+ signals (projection density) in the OT and PIR are encircled with white and yellow dashed lines, respectively. ***G***, ***H***, The 2D flatmaps constructed from five IUE@E11 (***G***) and seven IUE@E12 (***H***) individual mouse brains are shown. The numbers of mitral and tufted cells counted from five OB sections are listed under the maps. Dense GFP signals are observed throughout the majority of the olfactory cortex of IUE@E11 brains while only the anterior regions of IUE@E12 brains show dense GFP signals regardless of the numbers of labeled mitral and tufted cells. ***I***, The 2D flatmap in which the averaged IUE@E12 projection (***F***) was subtracted from the averaged IUE@E11 projection (***G***) to highlight the difference between two groups.

Next, we devised a digital flatmap of olfactory projection areas (e.g., olfactory cortices) to quantitatively and intuitively visualize the projection patterns. (Fig. 4*A–D*). The imaging registration to a common reference brain enabled us to create averaged projection patterns from each IUE@E11 and IUE@E12 brain. Figure 4*E*,*F* show the averaged distribution of GFP signals from IUE@E11 (*n* = 5) and IUE@E12 brains (*n* = 7), respectively. The flatmaps clearly indicate that the IUE@E12 brains send little to no projection to the posterior region of the olfactory cortex, such as the posterior PIR, ENTl, and amygdaloid cortex. This reflects the distribution patterns of the individual brain regardless of the numbers of labeled mitral and tufted cells (Fig. 4*G*,*H*). Previous studies have shown that the axons of tufted cells primarily project to the AON and OT (Igarashi et al., 2012; Hirata et al., 2019). Moreover, tufted and mitral cells preferentially project to the lateral and medial portion of the OT, respectively. Interestingly, our study shows that the density of GFP+ axons from the IUE@E12 brains, including the axons of late-generated mitral cells as well as those of tufted cells, project mostly to the lateral portion of the OT as compared with the broader projections from the IUE@E11 brains (Fig. 4*E*,*F*, regions encircled by white dashed lines). This result suggests that projections from late-generated mitral cells as well as tufted cells primarily innervate the lateral portion of the OT. In addition, the flatmap shows the density gradient of GFP+ axons from anterior to posterior PIR in the IUE@E12 brains (Fig. 4*F*, regions encircled by yellow dashed lines). The difference between the two groups is highlighted by subtracting the averaged IUE@E12 projection from the averaged IUE@E11 (Fig. 4*I*). We speculate that OB projection neurons may gradually shift their axonal endpoint from posterior to anterior within the PIR based on their birthdates.

## Discussion

### Topographically distinct projection patterns of early-generated and late-generated mitral cells

According to a recent study, a single progenitor cell is capable of giving rise to both mitral and tufted cells in the developing OB (Sánchez-Guardado and Lois, 2019). Nevertheless, the generation of mitral cells, which occur between E9–E13, is earlier than that of tufted cells, E11–E18 (Hinds, 1968; Hirata et al., 2019). These findings suggest that the timing of neurogenesis is a major determinant for the neuronal properties of OB projection neurons in the developing brain. Of particular interest is the fact that differences in birthdates among mitral cells or tufted cells result in the generation of OB projection neuron subpopulations with distinct cellular properties (Imamura et al., 2011; Imamura and Greer, 2015b; Hirata et al., 2019). This study demonstrated that the timing of neurogenesis also regulates the axonal projection pattern of different mitral cell subpopulations.

Our previous study showed that early-generated and late-generated mitral cell somata preferentially localized in dorsomedial and ventrolateral MCL, respectively (Imamura et al., 2011). Interestingly, the cortical amygdala receives afferent projections preferentially from mitral cells in the dorsomedial MCL (Miyamichi et al., 2011). Our current study demonstrated that late-generated mitral cells do not project to the posterior region of the olfactory cortex, and therefore it is likely that transmission of olfactory information from the dorsomedial OB to the cortical amygdala is mediated by early-generated mitral cells. This pathway may be essential for the mouse innate fear responses evoked by predator odors (Kobayakawa et al., 2007; Dewan et al., 2013; Root et al., 2014; Isosaka et al., 2015; Kondoh et al., 2016). On the other hand, the OT is innervated by mitral cells in the ventrolateral MCL as well as tufted cells (Scott et al., 1980; Imamura et al., 2011; Igarashi et al., 2012; Hirata et al., 2019). Our study also demonstrated that OB projection neurons generated around E12 innervate the lateral portion of the OT. It has been previously shown that an odor associated with punishment activates the lateral domain of the OT and induces aversive behavior while an odor associated with reward activates the anteromedial domain of the OT and induces attractive behavior (Murata et al., 2015; Yamaguchi, 2017; Zhang et al., 2017). Therefore, neural pathways from the OB to the OT may be mediated by distinct populations of OB projection neurons based on neuronal birthdates; i.e., early-generated OB projection neurons evoke attractive behavioral responses in mice, whereas late-generated OB projection neurons are responsible for aversive behaviors. Our study, therefore, suggests that birthdate-dependent mitral cell heterogeneity may be the origins of different olfactory information pathways.

One limitation to our study is that our *in utero* electroporation technique cannot directly discriminate the axons of late-generated mitral cells from those of tufted cells in the olfactory cortex, and therefore it is possible that late-generated mitral cells do not target the OT. However, we believe this to be unlikely based on our previous study using retrograde DiI labeling of OB projection neurons in which we concluded that mitral cells do innervate the OT (Imamura et al., 2011). This previous study also showed that more E12-generated mitral cells innervated the OT than E10-generated or E11-generated mitral cells. However, it was unknown whether the late-generated mitral cells project their axons to other regions of the olfactory cortex. Our current study clearly demonstrated that the late-generated mitral cells heavily project their axons to the anterior regions of the olfactory cortex, including the OT and AON, but not to the posterior regions. A critical next step is to reveal whether or not the cortical regions innervated by late-generated mitral cells are overlapped with those innervated by tufted cells.

### Methods to study the subsets of OB projection neurons

The *in utero* electroporation method has been widely used to label subpopulations of pyramidal neurons in a specific cortical layer as well as a specific type of retinal neurons that are generated at different embryonic days (Stancik et al., 2010; Matsuda, 2015; Bitzenhofer et al., 2017). This method is also effective to separately label OB projection neurons based on their birthdates. We have established an *in utero* method to target OB projection neurons and have further shown that the electroporation performed at different embryonic days introduces the plasmids into different subsets of mitral and tufted cells having different birthdates (Imamura and Greer, 2013, 2015b). Here, we performed the electroporation to introduce the GFP plasmids into mouse embryos at E11 and E12, and found that a significant number of mitral cells were labeled with GFP in the OB regardless of the electroporation timing. Although more GFP+ tufted cells were detected in the OBs following the E12 electroporation as compared with E11, a consistent finding with our previous study (Imamura and Greer, 2015b), a significant number of mitral cells were also labeled at E12 resulting in a mitral/tufted ratio of almost 1:1. Importantly, the mitral cells labeled with E12 electroporation were mostly the late-generated mitral cells.

On the other hand, separate labeling of the OB projection neurons having different birthdates has also been successfully accomplished by using a transgenic mouse line expressing CreERT2 under the Neurog2 promoter (Winpenny et al., 2011; Hirata et al., 2019). By altering the timing of tamoxifen injection into the Neurog2CreER x Cdhr1(Pcdh21)tTA x TREtdTomato mouse line (Hirata et al., 2019) induced expression of fluorescent markers in the OB projection neurons with different birthdates and analyzed their axonal projection patterns. They found that the tufted cells project their axons to the anterior regions of the olfactory cortex and that at least a subpopulation of external tufted cells, the last-generated OB projection neurons, innervates the anterolateral edge of the OT as well as the pars externa of the AON. However, unlike the previous report showing the enrichment of late-generated mitral cells in the ventrolateral OB (Imamura et al., 2011), the mitral cells labeled within the OB of this transgenic mouse were distributed in a random manner in the OB regardless of the time of tamoxifen injection. Thus, the *in utero* electroporation method may be more effective to segregate the early-generated and late-generated mitral cells.

### Generation of heterogeneity among OB projection neurons

The “canonical” mitral cell typically extends its secondary dendrites throughout the deep portion of the EPL. However, Orona et al. (1984) observed mitral cells with secondary dendrites extending in the intermediate portion of the EPL in the rat OB, although their somata laid in the MCL. Orona et al. (1984) classified mitral cells with secondary dendrites extending throughout the deep or intermediate EPL as Type I and Type II mitral cells, respectively. We have further revealed that early-generated and late-generated mitral cells extend their secondary dendrites in the deep and intermediate EPL, respectively, indicating that late-generated mitral cells can be classified as the previously identified Type II mitral cells (Imamura and Greer, 2015b). Combined with this study, the axonal projection of Type II mitral cells may localize to the more anterior regions of the olfactory cortex. Since the late-generated mitral cells possess the morphologic properties similar to those of tufted cells, an intriguing hypothesis is that the cellular properties of OB projection neurons are gradually shifted from mitral cells to internal tufted cells followed by middle and external tufted cells. In the developing OB, the progenitor cells may be programmed to produce projection neurons having slightly different properties throughout the course of neurogenesis. This might be a unique feature of the olfactory system since the cellular properties, especially the axonal projection patterns, of cortical pyramidal neurons generated at different timing seems to be less overlapped (Molyneaux et al., 2007; Gerfen et al., 2018).

In order to test the hypothesis that OB projection neuron diversity is derived from differences in neuronal birthdate, the molecular mechanisms underlying the generation of heterogeneity among the OB projection neurons must first be elucidated. Transcription factors play key roles in determining cellular phenotypes including fate, morphology, and molecular expression profile in developing cerebral pyramidal neurons (Kwan et al., 2012). To date, several transcription factors have been studied in this context with OB projection neurons, such as Tbr1, Tbr2, Neurog1, Neurog2, Sall1, Emx1, Pax6, and AP2ε (Yoshida et al., 1997; Bulfone et al., 1998; Arnold et al., 2008; Harrison et al., 2008; Feng et al., 2009; Shaker et al., 2012; Imamura and Greer, 2013). Of note, we reported that Tbr1 expression preceded Tbr2 in developing mitral cell (Imamura and Greer, 2013), suggesting that mitral cells follow a non-canonical pathway of differentiation in contrast to that described for cortical pyramidal neurons in which Tbr2 is expressed before Tbr1 during development (Englund et al., 2005). In addition, we and others demonstrated that each transcription factor appears in the developing OB with a distinct spatiotemporal pattern (Williams et al., 2007; Campbell et al., 2011; Nguyen and Imamura, 2019). Thus, comparing the types and time course of transcription factor expression among OB projection neurons generated at different time points during development is critical to understand the molecular mechanisms underlying the generation of OB projection neuron diversity. The results from large-scale analyses using omics approaches would help us to advance our knowledge in this field (Campbell et al., 2011; Kawasawa et al., 2016). The *in utero* electroporation method has the advantage of effectively modifying the molecular functions in a specific subset of mitral/tufted cells by introducing the plasmid vectors, and therefore can be used to study the function of transcription factors responsible for generating the birthdate-dependent differences among mitral cells.

In summary, this study demonstrated that late-generated OB projection neurons including late-generated mitral cells do not innervate the posterior regions of the olfactory cortex. In addition to somata location and dendritic distribution, our results suggest that the timing of neurogenesis also regulates the axonal projection patterns among OB projection neurons; not only between mitral and tufted cells but also among subpopulations of mitral cells.

## Synthesis

Reviewing Editor: Julie Bakker, University of Liege Decisions are customarily a result of the Reviewing Editor and the peer reviewers coming together and discussing their recommendations until a consensus is reached. When revisions are invited, a fact-based synthesis statement explaining their decision and outlining what is needed to prepare a revision will be listed below. The following reviewer(s) agreed to reveal their identity: Diego Restrepo, Michael Baum, Daniel Wesson.

Three reviewers have read the ms and all three are positive but do have some comments that need to be addressed.

Reviewer 1:

Mitral cells are heterogeneous in structure and function. This manuscript presents a study of axon projection of mitral/tufted (M/T) cells generated at days E11 or E12 (IUE@11 and IUE@12) in the mouse. The investigators introduce GFP in specific embryonic time points by electroporation of plasmids into Tbx21-Cre embryos restricting expression to mitral and tufted cells. They find that the IUE@12 cells, whose cell bodies are distributed in the ventrolateral olfactory bulb, project axons to the AON and anterior portion of the brain (AON and OT) while IUE@11 cells project to the entire olfactory cortex. This makes a substantial contribution to the understanding of the heterogeneity of mitral cells.

Major comments

1. Please provide a description of dendritic projections of the IUE@11 and IUE@12 cells. Were the findings consistent with the manuscript published in 2015?

2. The mice were sacrificed at P7. Did the authors perform a study of axon projection in older animals (say P14)? Do the projection patterns remain stable?

3. Was there a difference in the proportion of displaced mitral cells between IUE@11 and IUE@12 cells?

4. There appears to be a problem in displaying the average axonal projection signal in Figure 4F because it is very different from the individual mice shown in Figure 4H. In addition, it would help if the authors calculated a point by point ranksum p value corrected for multiple comparisons in order to understand the statistical differences between IUE@E11 and IUE@E12.

Minor comments

1. Please add axes in Figure 2A indicating the orientation of the slices (M-L, D-V).

2. Was the pattern of projection of IUE@E12 similar to the pattern of projection of the tdTomato axons?

Reviewer 2:

This manuscript describes an interesting study to determine whether slight differences in the fetal birth dates of neurons destined to become MOB mitral cells result in different axonal projection profiles of these mitral cells into different subdivisions of the mouse olfactory cortex. Using an elegant electroporation method for labeling newly born progenitor neurons destined to become MOB mitral cells and a sophisticated serial 2-photon tomography method to characterize mitral cell projection profiles in early adulthood, the investigators showed convincingly that mitral cells born/labeled on E12 projected exclusively to anterior olfactory cortex whereas mitral cells born/labeled a day earlier on E11 projected extensively over the entire olfactory cortex. The results, including histological examples, are nicely presented and quite convincing. The manuscript is generally well written, although it would still benefit from additional editing for minor mistakes in the use of English articles. My only major criticisms include: 1. The need for additional explanation of the authors’ decision to conduct in utero electroporation labeling on E11 vs E12. This is puzzling given that earlier research showed that MOB mitral cells can be born between E9 and E12. Why was such a brief, 1-day interval in labeling ages used in the present work? 2. The significance section of this manuscript essentially repeats the research results described in the Abstract. Likewise, the Discussion lacks any speculation about the functional (olfactory perception) significance of the authors’ present findings. The olfactory research community would pay more attention to the present results if the authors provided this information.

Reviewer 3:

This is a careful study wherein the authors explore principles governing the innervation of the olfactory cortex by olfactory bulb output neurons. How cortical innervation is dictated is of major importance for olfaction. Specifically, the authors probed how the date of birth of olfactory bulb output neurons may dictate the innervation of these neurons into olfactory cortical areas. The authors report that the birthdate does indeed segregate output neurons in terms of where they end up projecting. This study adds to other literature on how birthdates influences mitral and tufted cell body locations and dendritic projections by also showing how this imparts consequences on cortical innervation. The work is logical, well designed using a collection of careful modern methods, and the manuscript for the most part well written. I do have some concerns in need of handling.

Comments:

Introduction, last paragraph, the authors elude to knowledge from other papers that birthdate may influence projection patterns of mitral and tufted cells. The authors state that this “hasn’t been clearly demonstrated”. I’d appreciate the authors directly stating what was shown in those two studies so readers understand what is known. The authors are encouraged to be forthcoming here and not attempt to oversell significance since I believe it would be in everyone’s best interests to be factual and upfront regarding novelty and advance. The authors state in Discussion that they previously reported that OT receives preferential input from mitral cells depending upon date of birth. More introduction as to what is new is needed. If what is new is the quantification, this is novel enough in my mind, but it must be stated as such. Related to this, it seems a major weakness in this paper is the inability for the authors to disambiguate the projections of late birth date tufted versus mitral cells.

To me, this work holds some, albeit very different, similarities in spirit to that of John Scotts 1980 paper (The organization of projections from the olfactory bulb to the piriform cortex and olfactory tubercle in the rat). First, I think this paper should be cited. Second, I wonder if the authors could comment on one of the main take-home points of the Scott paper - that the ventral OB largely is what innervates the tubercle. Certainly the present injections weren’t restricted to select aspects of the bulb, but I wonder if any aspects of the present study support or confirm this notion? Are neurons in the ventral OB born at a different date than those in dorsal?

Minor comments:

Methods, I’m presuming the female mice were provided analgesia for pain relief before and/or following opening of the abdominal cavity. Please list.

Where is the optimal temperature cutting compound originating from?

Please provide catalog numbers for antibodies listed.

An RRID is included for DAPI and some of the mice but not the antibodies. Please insert RRIDs for the antibodies is available as otherwise done for the mice and DAPI.

Along which plane (coronal?) were the brains STPT imaged?

Fig 1B panels need some direction indicators and likely also lines to indicate cell layers to help readers appreciate what they are actually looking at. I gather the denser layer is the mitral cell layer, but indicating this would help. The authors are encouraged to include similar additions to improve appreciation for other panels as applicable.

The entire paper is well written, with exception of the abstract and significance statement. Just a few examples are listed below. Please correct the grammar in these sections.

-Abstract line 5: “mitral cells do consist of ...”. “do” may not be necessary in this statement

-Line 6, “are differentially localized their cell bodies"

-Line 11, “using the in utero electroporation method...” “the” and “method” are not necessary

-Line 16, “labeled with the E12", “the” is not needed. But moreover I’d suggest this statement could read instead as, “...late generated mitral cells do not extend...”

-The significance statement has other extraneous uses of “the” which the author could kindly remove to help readability

-The first sentence in the significance statement isn’t really useful since mitral cells and tufted cells form olfactory pathways, so how else would olfactory bulb pathways form? Please revise.

Finally, upon revision it would help reviewers for authors to have the page numbers listed consecutively, versus resetting on each page.

## Author Response

Dear Reviewers,

Thank you very much for reviewing our paper entitled “Topographically distinct projection patterns of early- and late-generated projection neurons in the mouse olfactory bulb” (by Chon, LaFever, Nguyen, Kim, and Imamura). We have worked hard to ensure that all of the comments we received from the reviewers have been fully addressed. The comments and recommendations from the reviewers were constructive and we hope that you will agree that incorporation of these into a revised manuscript would be appropriate and result in a much stronger submission to eNeuro. The responses to each of the recommendations are detailed below:

Reviewer 1

This makes a substantial contribution to the understanding of the heterogeneity of mitral cells.

We thank the Reviewer 1 for the very positive comments on the significance of our work.

Major comments:

1. Please provide a description of dendritic projections of the IUE@11 and IUE@12 cells. Were the findings consistent with the manuscript published in 2015?

Thank you very much for your suggestion. In this study, we confirmed that the GFP+ secondary dendrites were preferentially distributed in the superficial EPL in the IUE@E12 OB, which can be seen in Figure 2A and is consistent with the previous study (Imamura and Greer, 2015b). We have added the description in the Results of our revised manuscript.

2. The mice were sacrificed at P7. Did the authors perform a study of axon projection in older animals (say P14)? Do the projection patterns remain stable?

Although the OB projection neuron-specific expression of GFP from the PCALNL-GFP plasmid electroporated into the Tbx21-Cre mice were confirmed at P7 (Fig. 1A, B), the study of axonal projection pattern using the STPT imaging (Fig. 1C, D, 2, 3, and 4) was performed with the mice sacrificed between 6 and 8 weeks old (P42 - P53). We apologize for the unclear description. We have modified the sentences in the Results of our revised manuscript to make this point clearer.

3. Was there a difference in the proportion of displaced mitral cells between IUE@11 and IUE@12 cells?

This is an interesting point. Upon our observation of slices of IUE@E11 and IUE@E12 OBs, we have the impression that there were more mitral cells localized superficial portion of the MCL in the IUE@E12 OBs; implying that more displaced mitral cells were labeled with IUE@E12 compared to IUE@E11. However, since there is no good criterion to discriminate displaced mitral cells from other mitral cells, we could not perform any quantitative analysis to show a difference in the proportion of displaced mitral cells between IUE@E11 and IUE@E12. Therefore, we have decided not to describe the proportion of displaced mitral cells in the manuscript. We hope that the reviewer would agree with our decision.

4. There appears to be a problem in displaying the average axonal projection signal in Figure 4F because it is very different from the individual mice shown in Figure 4H.

We thank the reviewer very much for pointing this out. We double-checked the steps to display our data in 2D flatmaps shown in Figure 4 and found that there was an error in a step to color bins in the flatmaps of the individual mice (old Figure 4H and I). Each bin in a flatmap should have been colored based on the percentages of GFP+ pixels among total pixels (projection density) in each bin. However, in the flatmaps shown in old Figure 4H and I, after the projection density was calculated for each bin, the bin was mistakenly colored based on the percentages of the projection density value among the sum of the projection density values of all bins in the flatmap. We sincerely apologize for the error and have replaced the wrong flatmaps (old Figure 4H and I) with the correct ones (new Figure 4G and H) in Figure 4.

In addition, it would help if the authors calculated a point by point ranksum p value corrected for multiple comparisons in order to understand the statistical differences between IUE@E11 and IUE@E12.

This is another interesting point. According to the suggestion, we ran a statistical analysis to see a significant difference in our flatmap data between the two groups. However, none of the bins in the flatmap came out as significant after multiple comparison corrections largely due to variabilities in the number and location of labeled cells from the two groups. As an alternative way to highlight our overall conclusion that IUE@E11 showed broader projection reaching to the caudal part of olfactory cortices compared to IUE@E12, we subtracted the averaged IUE@E12 projection from the averaged IUE@E11 projection and add a new Figure 4I and a description. We hope that the reviewer would agree that the figure is enough to support our conclusion.

Minor comments:

1. Please add axes in Figure 2A indicating the orientation of the slices (M-L, D-V).

Thank you very much for your suggestion. The axes indicating the orientation of the slices have been added in Figure 2A.

2. Was the pattern of projection of IUE@E12 similar to the pattern of projection of the tdTomato axons?

To analyze the axonal projection patterns of OB projection neurons generated at different developmental stages, we electroporated pCALNL-GFP into the brains of Tbx21Cre x tdTomato transgenic mice. Since all mitral and tufted cells express tdTomato in Tbx21Cre x tdTomato mice, tdTomato+ axons were seen in the entire olfactory cortex and the projection pattern was not similar to the GFP+ axons in the IUE@E12 brain. We apologize for the unclear description. To make this point clearer, we have modified the sentences in the Results of our revised manuscript as follows; “we electroporated pCALNL-GFP into the brains of Tbx21Cre x tdTomato transgenic mice. In these mice, tdTomato is expressed by all OB projection neurons (Nguyen and Imamura, 2019)”.

Reviewer 2

The results, including histological examples, are nicely presented and quite convincing. The manuscript is generally well written, although it would still benefit from additional editing for minor mistakes in the use of English articles.

We thank the Reviewer 2 for the very positive comments. We have asked a native English speaker to double-check the grammar in the manuscript.

1. The need for additional explanation of the authors’ decision to conduct in utero electroporation labeling on E11 vs E12. This is puzzling given that earlier research showed that MOB mitral cells can be born between E9 and E12. Why was such a brief, 1-day interval in labeling ages used in the present work?

Thank you very much for making an important point. Our previous studies showed that differences in cell body location and dendrite extension pattern between E11- and E12-generated mitral cells were greater than those between E10- and E11-generated mitral cells (Imamura et al., 2011; Imamura and Greer, 2015). Based on these findings, we assumed that E12-generated mitral cells significantly change their cellular properties from E11-generated mitral cells. Therefore, we decided to conduct in utero electroporation labeling on E11 and E12 to examine whether there is a birthdate-dependent difference in the axonal projection patterns in this study. The description to justify our decision to conduct in utero electroporation labeling on E11 vs E12 has been added to the Results section of the revised manuscript.

2. The significance section of this manuscript essentially repeats the research results described in the Abstract.

We appreciate your constructive comment. We rewrote the Significance section. We hope that the reviewer would agree that the new paragraph is not the repeats of the Abstract, is describing the significance of this study, and is appropriate for the Significance section.

Likewise, the Discussion lacks any speculation about the functional (olfactory perception) significance of the authors’ present findings. The olfactory research community would pay more attention to the present results if the authors provided this information.

Another excellent suggestion. Our previous study showed that early- and late-generated mitral cells preferentially localized in dorsomedial and ventrolateral MCL, respectively (Imamura et al., 2011), and this study demonstrated that the late-generated mitral cells restrict their axonal projections solely to the anterior regions of the olfactory cortex. In addition, it was shown that the cortical amygdala and OT receive afferent projections preferentially from mitral cells in the dorsomedial and ventrolateral MCL, respectively (Miyamichi et al., 2010; Scott et al., 1980; Imamura et al., 2011; Igarashi et al., 2012; Hirata et al., 2019). Therefore, based on our findings, we can speculate that the olfactory information pathways originating from early- and late-generated mitral cells are essential for the animal behaviors regulated by the cortical amygdala and OT, respectively. For example, it is known that the cortical amygdala is necessary for the mouse innate fear responses evoked by predator odors (Kobayakawa et al., 2007; Dewan et al., 2013; Root et al., 2014; Isosaka et al., 2015; Kondoh et al., 2016), while the OT is suggested to be involved in the attractive and aversive behaviors elicited by specific odors (Murata et al., 2015; Yamaguchi, 2017; Zhang et al., 2017). We have added a paragraph describing above our speculation about the functional significance of our present findings in the Discussion of the revised manuscript.

Reviewer 3

This study adds to other literature on how birthdates influences mitral and tufted cell body locations and dendritic projections by also showing how this imparts consequences on cortical innervation. The work is logical, well designed using a collection of careful modern methods, and the manuscript for the most part well written.

We thank the Reviewer 3 for acknowledging the importance of our work and positive comments on the manuscript.

Comments:

Introduction, last paragraph, the authors elude to knowledge from other papers that birthdate may influence projection patterns of mitral and tufted cells. The authors state that this “hasn’t been clearly demonstrated”. I’d appreciate the authors directly stating what was shown in those two studies so readers understand what is known. The authors are encouraged to be forthcoming here and not attempt to oversell significance since I believe it would be in everyone’s best interests to be factual and upfront regarding novelty and advance.

The authors state in Discussion that they previously reported that OT receives preferential input from mitral cells depending upon date of birth. More introduction as to what is new is needed. If what is new is the quantification, this is novel enough in my mind, but it must be stated as such.

We appreciate the reviewer’s constructive suggestions. Regarding the relationship between birthdate and axonal projection pattern of OB projection neurons, previous studies demonstrated the OT receives axonal inputs preferentially from tufted and late-generated mitral cells (Scott et al., 1980; Imamura et al., 2011), and segregated axonal projections are formed by early-generated mitral cells and late-born external tufted cells (Hirata et al., 2019). Nevertheless, the axonal projection of late-generated mitral cells to the olfactory cortex other than the OT, and differences in axonal projection patterns between early- and late-generated mitral cells have not yet been elucidated. A novelty of our study is that we demonstrate, for the first time, that not only tufted cells but also late-generated mitral cells restrict their axonal projections solely to the anterior regions of the olfactory cortex. We have added the description in the last paragraph of the Introduction of the revised manuscript.

Related to this, it seems a major weakness in this paper is the inability for the authors to disambiguate the projections of late birth date tufted versus mitral cells.

We believe that our study clearly shows that late-generated mitral cells restrict their axonal projections solely to the anterior regions of the olfactory cortex and, therefore, there is a difference in axonal projection patterns between early- and late-generated mitral cells. However, we admit that the inability to disambiguate the projections of late-generated mitral cells versus tufted cells is a weakness of our study. Segregating the axonal projection of late-generated mitral cells from tufted cells would further advance our knowledge in olfactory information pathways. However, we hope that the reviewer agrees with our thought that it is a scope of future studies. We have included these points in the Discussion of our manuscript.

To me, this work holds some, albeit very different, similarities in spirit to that of John Scotts 1980 paper (The organization of projections from the olfactory bulb to the piriform cortex and olfactory tubercle in the rat). First, I think this paper should be cited. Second, I wonder if the authors could comment on one of the main take-home points of the Scott paper - that the ventral OB largely is what innervates the tubercle. Certainly the present injections weren’t restricted to select aspects of the bulb, but I wonder if any aspects of the present study support or confirm this notion? Are neurons in the ventral OB born at a different date than those in dorsal?

Another constructive suggestion. As pointed out by Reviewer 3, Dr. Scott and his colleagues showed that the OT receives heavier axonal inputs from the ventrolateral OB (Scott et al., 1980). In addition, our previous study showed that early- and late-generated mitral cells preferentially localized in dorsomedial and ventrolateral MCL, respectively (Imamura et al., 2011). We have cited these papers and added paragraphs in Discussion to discuss our speculation about the functional significance of our finding that the late-generated mitral cells localize preferentially in the ventrolateral MCL and project their axons only to the anterior portion of the olfactory cortex. Please see our response to Reviewer 2 for more details.

Minor comments:

Methods, I’m presuming the female mice were provided analgesia for pain relief before and/or following opening of the abdominal cavity. Please list.

Description of analgesia, “The animals were given a subcutaneous injection of Carprofen (5mg/kg) for pain relief before and after the surgery.”, has been added to the in utero electroporation section of Materials and Methods.

Where is the optimal temperature cutting compound originating from?

The optimal temperature cutting compound was originated from the Sakura Fintek USA. We have added the information in the Material and Methods on our revised manuscript.

Please provide catalog numbers for antibodies listed.

An RRID is included for DAPI and some of the mice but not the antibodies. Please insert RRIDs for the antibodies is available as otherwise done for the mice and DAPI.

We have added the information of mice, antibodies and plasmids, Catalog# and/or RRID, in the Materials and Methods of our revised manuscript.

Along which plane (coronal?) were the brains STPT imaged?

The STPT imaging was performed along the coronal plane. The information has been included in the Materials and Methods of our revised manuscript.

Fig 1B panels need some direction indicators and likely also lines to indicate cell layers to help readers appreciate what they are actually looking at. I gather the denser layer is the mitral cell layer, but indicating this would help. The authors are encouraged to include similar additions to improve appreciation for other panels as applicable.

Thank you very much for your suggestion. Figure 1B is an image taken from the medial region of a coronal section of P7 Tbx21-Cre OB electroporated with pCALNL-GFP and pCAG-tdTomato, at E11. According to the suggestion, we have added the description to explain that the image was taken from the medial region in Figure Legend and added the lines to indicate cell layers in Figure 1B. We also have added the axes indicating the orientation of the slices or brain images in Figure 1D1, 2A1, 2D1, 3B7, and 3C1.

The entire paper is well written, with exception of the abstract and significance statement. Just a few examples are listed below. Please correct the grammar in these sections.

-Abstract line 5: “mitral cells do consist of ...”. “do” may not be necessary in this statement

-Line 6, “are differentially localized their cell bodies"

-Line 11, “using the in utero electroporation method...” “the” and “method” are not necessary

-Line 16, “labeled with the E12", “the” is not needed. But moreover I’d suggest this statement could read instead as, “...late generated mitral cells do not extend...”

Thank you very much for correcting the grammatical errors. We have corrected the errors accordingly.

-The significance statement has other extraneous uses of “the” which the author could kindly remove to help readability

-The first sentence in the significance statement isn’t really useful since mitral cells and tufted cells form olfactory pathways, so how else would olfactory bulb pathways form? Please revise.

Thank you very much for your suggestion. According to Reviewer 2 and your suggestions, the significance section has been rewritten. Please also see our response to Reviewer 2.

Finally, upon revision it would help reviewers for authors to have the page numbers listed consecutively, versus resetting on each page.

Thank you very much for your suggestion. In the revised manuscript, we have consecutively listed the line numbers across the pages.
